# Acute disseminated encephalomyelitis following Influenza A pneumonia

**DOI:** 10.1002/ccr3.1353

**Published:** 2017-12-22

**Authors:** Babikir Kheiri, Emad Abu Sitta, Anas Salih, Mohammed Al Qasmi, Ghassan Bachuwa

**Affiliations:** ^1^ Internal Medicine Department Hurley Medical Center/Michigan State University Two Hurley Plaza, Ste 212 Flint Michigan 48503

**Keywords:** Acute disseminated encephalomyelitis, autoimmune demyelinating disorder

## Abstract

Acute disseminated encephalomyelitis (ADEM) is an autoimmune demyelinating disorder of the central nervous system and can present following influenza A infections as multifocal neurological deficits. ADEM remains a challenging diagnosis, and high clinical suspicious coupled with laboratory investigations and neuroimaging is required to exclude other primary and secondary demyelinating disorders.

## Introduction

Influenza A pneumonia can be associated with adulthood acute disseminated encephalomyelitis (ADEM). ADEM remains a challenging diagnosis as the lack of specific disease biomarkers; therefore, high clinical judgment coupled with laboratory investigations and neuroimaging is required to exclude other primary and secondary demyelinating diseases.

## Quiz Question

What are the investigations and what is the diagnosis?

## Case Presentation

**Figure 1:**
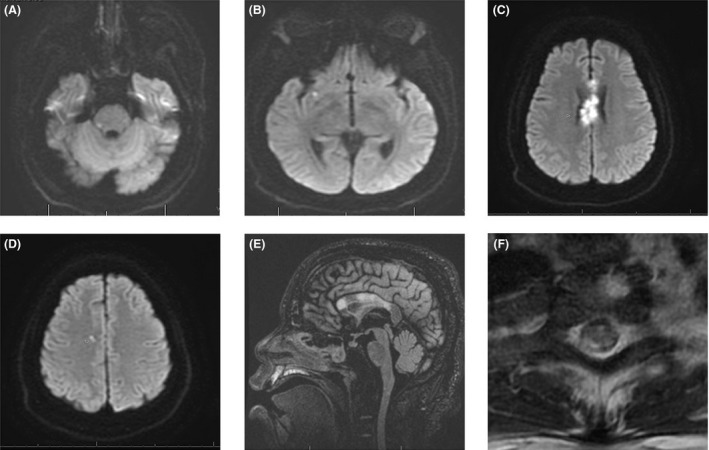
MRI without gadolinium. (A–D) Axial DWI MRI showing diffusion limited restricted diffusion involving right posterolateral aspect, right temporal region, corpus callosum, and right central semiovale. In addition, corpus callosum showing expansive lesions. There was also associated T2‐weighted and fluid‐attenuated inversion recovery (FLAIR) hyperintensities. (E) Sagittal FLAIR MRI showing hyperintense signals involving corpus callosum. (F) Axial T2‐weighted MRI cervical spine showing hyperintense signals involving posterior columns of cervical cord at C2 level.

**Figure 2:**
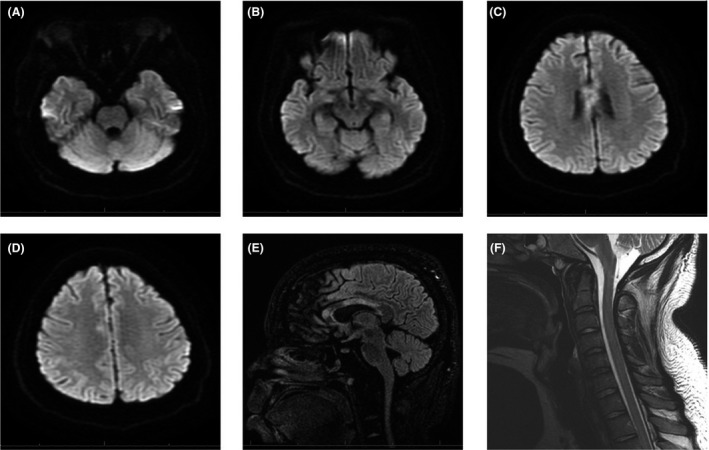
MRI images without gadolinium showing decreased signals in comparison with Fig. 1.

A 29‐year‐old adult with past medical history of insulin‐dependent diabetes mellitus, hypertension, and chronic kidney disease (CKD) was admitted with acute respiratory failure secondary to ARDS. He tested positive for influenza A. He then developed progressive generalized weakness after 2 weeks. An urgent CT head without contrast showed no acute intracranial process. His condition worsened dramatically over the following 2 weeks, and he became quadriplegic. Neurological examination revealed normal cranial nerve testing, quadriparesis, and intact sensation and reduced tone and reflexes for all four limbs. Brain magnetic resonance imaging (MRI) without gadolinium, given his CKD, showed multiple asymmetrical patchy and punctiform hyperintensities within corpus callosum (Fig. 1). In addition, a cervical MRI showed hyperintense signals involving the posterior columns of cervical cord at C2 vertebral level (Fig. 1F). Cerebrospinal fluid (CSF) analysis revealed elevated protein at 157 mg/dl and elevated glucose 158 mg/dl, with normal WCC, RBC, and negative culture. Additional CSF testing was negative for oligoclonal bands, VDRL, and paraneoplastic autoantibodies panel. Secondary causes of demyelinating CNS disorders including vasculitis, Lyme disease, human immunodeficiency virus, Devic's disease/neuromyelitis optica (NMO), and neurosarcoidosis were excluded. He was started on intravenous methylprednisolone at dose of 1 g/day for 5 days followed by oral prednisone taper. Over the following few weeks, his upper limbs weaknesses have moderately improved; however, he still has residual lower limbs weakness. Few months later, a repeated MRI was performed and showed interval slightly decreased abnormal signals (Fig. 2).

ADEM is a postinfectious/vaccination autoimmune demyelinating disorder involving the central nervous system (CNS) and manifests commonly as monophasic disease in children 1. However, multiphasic forms (relapsing ADEM) and adult presentations have been identified 1. Seasonal and pandemic influenza infections have been linked to several rare neurological complications including ADEM, often with pandemic influenza H1N1 infection 2. ADEM diagnosis requires exclusion of other demyelinating diseases in patients with presumed clinical inflammatory demyelinating CNS event, encephalopathy, and specific brain abnormalities 1.

MRI is paramount in excluding primary (multiple sclerosis and neuromyelitis optica) and secondary causes of CNS demyelinating diseases and has been shown to be very sensitive in detecting the white matter abnormalities associated with ADEM [3]. Typically, T2‐weighted and FLAIR sequences demonstrate multiple asymmetrical patch and poorly marginated bilateral hyperintense lesions with variable sizes, and involve the subcortical/central white matter with periventricular sparing, cortical gray–white matter junction of cerebellum and brainstem, and the gray matter of the thalami and basal ganglia, with variable gadolinium enhancement 1. Involvement of the spinal cord usually described as large confluent intramedullary lesions extending over several segments with variable gadolinium enhancement and sometimes associated with cord swelling 1.

## Conflict of Interest

None declared.

## Authorship

BK: designed, planned, wrote the manuscript, and did the literature review. EA: designed, planned, and revised the manuscript. AS: designed, planned, revised the manuscript, and prepared the photographs. OA: designed, planned, and revised the manuscript. GB: designed, planned, and revised the manuscript.
